# Supplementary material for: Predicting visual function by interpreting a neuronal wiring diagram
Source: Nature. 2024 Oct 2;634(8032):113–23. doi: 10.1038/s41586-024-07953-5 (PMC11446822; doi:10.1038/s41586-024-07953-5)
Supplement: Supplementary file 6 — CRF and ERF predictions for individual TmY4 and TmY9 cells. Analogous to Supplementary Data 3, but for TmY target types. Shown are the top four monosynaptic pathways, the strongest pathway passing through each of the top ten intermediary types (ranking from Extended Data Fig. 7), and the trisynaptic pathway Tm1–TmY–Dm3–TmY (see the section entitled Prediction of spatial normalization). [file 41586_2024_7953_MOESM6_ESM.zip › DataS4/TmY9qΓèÑ/720575940632385361.pdf]

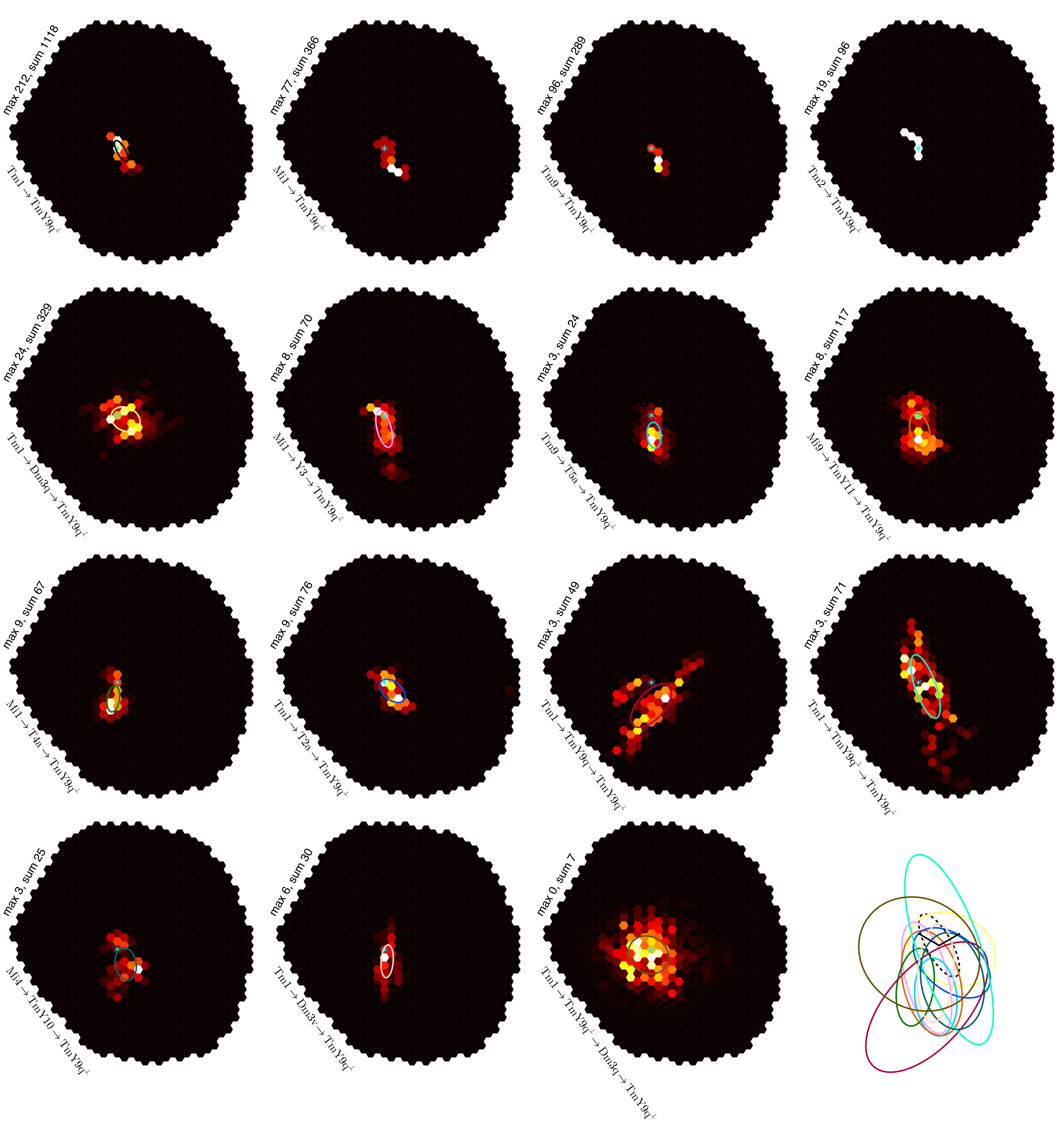

max 212, sum 1118
Tm1 → TmY9q⊥
max 77, sum 366
Mi1 → TmY9q⊥
max 96, sum 289
Tm9 → TmY9q⊥
max 19, sum 96
Tm2 → TmY9q⊥
max 24, sum 329
Tm1 → Dm3q → TmY9q⊥
max 8, sum 70
Mi1 → Y3 → TmY9q⊥
max 3, sum 24
Tm9 → T5a → TmY9q⊥
max 8, sum 117
Mi9 → TmY11 → TmY9q⊥
max 9, sum 67
Mi1 → T4a → TmY9q⊥
max 9, sum 76
Tm1 → T2a → TmY9q⊥
max 3, sum 49
Tm1 → TmY9q → TmY9q⊥
max 3, sum 71
Tm1 → TmY9q⊥ → TmY9q⊥
max 3, sum 25
Mi4 → TmY10 → TmY9q⊥
max 6, sum 30
Tm1 → Dm3v → TmY9q⊥
max 0, sum 7
Tm1 → TmY9q⊥ → Dm3q → TmY9q⊥
